# Melatonin to treat sleep disturbances in patients with Alzheimer’s disease

**DOI:** 10.1002/alz.083775

**Published:** 2025-01-09

**Authors:** Viacheslav Viktorovich Sushko, Viktor Vasilievich Sushko

**Affiliations:** ^1^ National University “Odessa Law Academy”, Odessa Ukraine; ^2^ Odessa National Maritime University, Odessa Ukraine

## Abstract

**Background:**

Alzheimer’s disease can cause sleep disturbances in humans, which can worsen other symptoms of the disease.

**Method:**

In our study, we examined the sleep patterns of 23 patients with Alzheimer’s disease, aged 65‐74 years (20 women and 3 men), over 4 months. All patients reported experiencing poor sleep, including difficulty sleeping in the ward, frequent awakenings during the night, early morning awakenings, and daytime sleepiness. To assess their sleep, we conducted a polysomnographic study in a specialized ward with continuous monitoring of indicators, video, and audio control. This was done both before the patients were included in the study and after a month of melatonin administration. During the diagnostic night, a nurse was present to continuously observe the polysomnography process. Based on the results of the polysomnography, we constructed a hypnogram, which is a curve that depicts the quality of sleep, the number and duration of sleep stages, and their structure. The patients were given a dose of 1.5 mg of melatonin (half a tablet) 30 minutes before bedtime for 4 weeks, with gradual withdrawal.

**Result:**

In the initial polysomnographic study, all patients showed shortened phases of rapid eye movement (REM) and slow eye movement (SEM), with a rapid transition between them during sleep. They also experienced long periods before falling asleep and 3 to 4 nocturnal awakenings. However, after a month of melatonin administration, the majority of patients showed a normalization of REM and SEM phases, in line with their age norms, and an improvement in the duration of sleep. The number of nocturnal awakenings also decreased to 1‐2, mostly between 4 and 6 hours. Additionally, patients were able to fall back asleep after awakening, and the time it took for them to fall asleep decreased. However, even after a month of melatonin supplementation, some patients still experienced early morning awakenings and daytime sleepiness and reported feeling unrefreshed after a night’s sleep.

**Conclusion:**

In conclusion, our study suggests that melatonin is effective in treating sleep disorders in patients with Alzheimer’s disease, and can help normalize the duration and frequency of REM and SEM phases during sleep.

